# PHYSICAL ACTIVITY AND SERUM BIOMARKERS IN OLDER WOMEN WITH CARDIOVASCULAR DISEASE EXPERIENCING FATIGUE

**DOI:** 10.1093/geroni/igae098.3287

**Published:** 2024-12-31

**Authors:** Shamatree Shakya, Nathan Tintle, Lisa Sharp, Shannon Halloway

**Affiliations:** University of Illinois Chicago, Chicago, Illinois, United States; University of Illinois Chicago, Chicago, Illinois, United States; University of Illinois Urbana-Champaign, Champaign, Illinois, United States; University of Illinois Chicago, Chicago, Illinois, United States

## Abstract

Fatigue is a common symptom of cardiovascular disease (CVD) and can be an early indicator of frailty. Physical activity (PA) can improve fatigue and frailty-related serum biomarkers: brain-derived neurotrophic factor (BDNF), vascular endothelial growth factor-A (VEGF A), and insulin-like growth factor-1 (IGF-1). However, variation in PA and serum biomarkers among patients with CVD who are experiencing fatigue is understudied. We aimed to investigate (a) differences in PA and serum biomarkers in women with CVD experiencing fatigue and (b) the influence of PA on fatigue. We used cross-sectional, baseline data from older women (≥ 65 years, N=253) with CVD participating in the MindMoves trial (NCT04556305). PA included ActiGraph accelerometer measures of sedentary behavior, light PA, moderate-to-vigorous PA, and daily step count, and cardiorespiratory fitness. Serum biomarkers included BDNF, VEGF-A, and IGF-1. Fatigue was assessed using two items from the Center for Epidemiologic Studies-Depression Scale. Two-sample t-tests examined differences in PA and biomarkers across subgroups with and without fatigue. Logistic regression was used to examine associations between significant PA variables and fatigue. Fatigue was prevalent in 17% of participants. There were significantly fewer daily minutes of MVPA (p=0.0005) and lower daily step count (p=0.0142) in those experiencing fatigue. No significant differences were observed in serum biomarkers. After adjusting for sociodemographic characteristics, no PA variable was associated with fatigue. Women with CVD who experience fatigue had lower engagement in PA. Future research is needed to investigate fitness measures (e.g., strength) and other biomarkers to understand underlying physiological processes influencing PA in fatigue.

